# Long-term potentiation and neurotransmitters expression and segregation are altered in the Metabolic Syndrome-associated dysautonomia

**DOI:** 10.1371/journal.pone.0335728

**Published:** 2025-11-04

**Authors:** Diana Elinos, Fernanda Veladiz-Gracia, Constanza González-Sierra, Angel Rubio-Galicia, Fredy Cifuentes, Miguel A. Morales

**Affiliations:** Departamento de Biología Celular & Fisiología, Instituto de Investigaciones Biomédicas, Universidad Nacional Autónoma de México, Ciudad de México, México; Shiga Medical Center, JAPAN

## Abstract

The autonomic nervous system (ANS) dynamically regulates the internal environment to maintain homeostasis. The ANS exhibits some forms of synaptic plasticity, including long-term potentiation (LTP) and plastic changes in neurotransmitter distribution, both of which may contribute to autonomic function. Dysautonomia refers to an abnormality in the function of the ANS, with an imbalance between sympathetic and parasympathetic activity. Dysautonomia has been reported in conditions such as stress, hypertension, and metabolic syndrome (MS). MS is a cluster of risk factors for cardiovascular disease, diabetes, and premature death. In MS, the signs of dysautonomia include elevated plasma norepinephrine levels and increased arterial blood pressure. In this study, we characterized the effect of a high-sucrose diet (HSD) on synaptic plasticity in sympathetic ganglia of the rat by measuring LTP expression in the superior cervical ganglion (SCG) and analyzing the expression of acetylcholine (ACh) and GABA, as well as their balance of colocalization/segregation in ganglionic nerve terminals. The HSD consists of adding 30% sucrose to drinking water, which is an accepted model of MS. We observed an impairment in LTP expression, along with a decrease in ACh presence and a reduction in its segregation from GABA. These findings suggest the emergence of an inhibitory effect on synaptic transmission and plasticity within the SCG. We propose that dysautonomia associated with MS might involve changes in sympathetic activity, at least at the level of ganglionic cholinergic transmission. These results may help to improve our understanding of autonomic dysfunction in the context of this metabolic disorder.

## Introduction

The autonomic nervous system (ANS) composed of the sympathetic, parasympathetic, and enteric divisions, regulates various physiological processes, including heart rate, blood pressure, metabolism [1]. These functions are dynamically adjusted throughout the day to meet the physiological demands of the organism, during rest, stress, or exercise.

The ANS exhibits various forms of synaptic plasticity that may contribute to the control and regulation of target organs. One form of synaptic plasticity in the sympathetic system is ganglionic long-term potentiation (gLTP), a sustained increase in synaptic efficacy observed in sympathetic ganglia, such as the superior cervical ganglion (SCG) [2–4]. Another form of plasticity involves changes in neurotransmitter expression, localization, and merging. Previous studies have demonstrated that these plasticity mechanisms occur in the SCG of rats at different ages and in hypertension [5–7].

Dysautonomia, or autonomic dysfunction, refers to impairments in ANS function, manifested as an imbalance between sympathetic and parasympathetic activity, either through reduced or excessive function. Dysautonomia may be present as a primary disorder or as a result of other pathologies. It has been reported in conditions such as stress [5], hypertension (HT) [4], diabetes [8,9], and metabolic syndrome (MS) [10,11].

Autonomic function can be assessed in both the sympathetic and parasympathetic divisions, either directly or indirectly, using various methods. Sympathetic activity can be evaluated by measuring plasma or urinary norepinephrine (NE) levels, recording muscle sympathetic nerve activity (MSNA), and monitoring blood pressure. Parasympathetic function can be assessed through heart rate responses to postural changes and baroreflex sensitivity. Additionally, heart rate variability analysis serves as an indicator of both autonomic components [1,9,12,13]. We have proposed the study of activity in specific efferent pathways as an alternative approach to characterizing changes in sympathetic function. In the SCG, we have characterized basal synaptic transmission, gLTP, and neurotransmitter expression and distribution under various conditions. Previous studies have shown that in certain pathologies involving dysautonomia, sympathetic plasticity is altered. For instance, increased basal synaptic transmission and gLTP expression have been observed in spontaneously hypertensive rats (SHR) [4], along with changes in neurotransmitter expression and segregation in response to stress [5], HT [4], and aging [7].

The sympathetic nervous system (SNS) also plays a role in metabolic regulation by controlling energy expenditure, modulating resting metabolic rate, and initiating thermogenesis in response to relevant stimuli [14]. MS is a cluster of risk factors that increase the likelihood of developing cardiovascular disease, type 2 diabetes, and premature mortality. These risk factors include abdominal obesity, low HDL cholesterol levels, hypertriglyceridemia, elevated blood pressure, and fasting hyperglycemia. A diagnosis of MS is made when abdominal obesity is present along with at least two of the other risk factors [15].

Changes in sympathetic activity are thought to contribute to the pathophysiology of MS. Evidence from studies on HT and obesity shows that increased sympathetic drive is associated with tachycardia, elevated plasma NE levels, and increase sympathetic nerve activity [16]. Moreover, excessive sympathetic activity has been implicated in elevated arterial blood pressure and impaired glucose metabolism in both humans and animal models of MS [17]. In contrast, other studies have reported depressed synaptic transmission in the SCG and celiac ganglion of streptozotocin (STZ)-treated mice [8,18]. Therefore, assessing sympathetic function is critical for improving MS diagnosis, and treatments aimed at restoring sympathetic-parasympathetic balance may offer a therapeutic approach [11,16].

To contribute to the characterization of MS-associated dysautonomia, we used a high-sucrose diet protocol that induces changes in metabolic parameters such as glycemia, triglyceride levels, peri-abdominal fat accumulation, and hepatic steatosis. To further assess dysautonomia, we advance in characterizing synaptic plasticity by measuring gLTP expression, neurotransmitter presence, and the balance between the coexistence and segregation of acetylcholine (ACh) and GABA in the SCG of rats. Preliminary findings have been published in abstract form [19].

## Materials and methods

### Animals

Standard male Wistar rats, 8 weeks old, 250–300 g, were used. In total 16 animals were used, 8 experimental and 8 control. They were handled in accordance with the ethical guidelines for the care and use of laboratory animals of the National Academy of Sciences of the United States. The project was approved by the Committee for the Care and Use of Laboratory Animals (CICUAL) of our Institute. Every effort was made to minimize the number of animals used, as well as the stress in handling them. Animals were housed in a designed room laboratory with controlled 22 °C temperature and a 12/12 light-dark cycle starting light period at 7:00 hrs. Animals were euthanized by deep anesthesia with sodium pentobarbital (125 mg/kg, i.p.), followed by perfusion with a fixative solution.

### Induction of the experimental model

Metabolic syndrome (MS) was induced by a chronic high-sucrose diet (HSD), supplying the drinking water with 30% (w/v) sucrose [20]. This HSD regimen was maintained for 8 weeks during which weight, food and water intake were recorded. During this period, control group has standard chow diet (Teklad 2018S, Inotiv, Madison, WI, USA) and tap water *ad libitum*.

### Metabolic parameters

Weight and glycemia were measured in unanesthetized rats. After anesthesia (ketamine, 90 mg/kg, and xylazine hydrochloride 10 mg/kg i.p.) and SCG dissection for electrophysiology, animals were deeply anesthetized with sodium pentobarbital (125 mg/kg i.p.). Blood was then collected via cardiac puncture, centrifuged to obtain serum, and stored at −20°C for later analysis of glucose, cholesterol, and triglycerides. Following blood collection, rats were transcardially perfused with ice-cold phosphate-buffered saline (0.01 M PBS, pH 7.4), followed by an ice-cold fixative solution (4% paraformaldehyde in 0.1 M PBS, pH 7.4). The liver was retrieved and postfixed in the same fixative solution for 2 hours. Additionally, retroperitoneal fat was removed and weighed.

### Lipid staining

Oil Red O staining was used to detect hydrophobic lipids (cholesteryl esters and triglycerides) in hepatocytes. A section of fixed liver tissue was cryoprotected in a 30% sucrose solution, frozen at −20°C, and cut into 10 µm-thick sections using a cryostat (Leica CM1520, Wetzlar, Germany). The sections were mounted on gelatin-coated Superfrost slides (Electron Microscopy Sciences, Hatfield, PA, USA) and washed in 0.1 M PBS for 5 minutes, followed by a 5-minute wash in 100% propylene glycol. Next, the sections were stained by immersion in a freshly prepared, warm 0.5% Oil Red O solution (Sigma-Aldrich, St. Louis, MO, USA) in propylene glycol for 30 minutes. After staining, the sections were washed again in 100% propylene glycol for 5 minutes, counterstained with Harris hematoxylin, and mounted using Vector mounting medium (Vector Laboratories, Newark, CA, USA). Images were captured using a digital camera COMS Jenoptik ProgRes GRYPHAX (Jenoptik AG, Jena, Germany) attached to a Nikon Optiphot-2 microscope (Nikon Corporation, Tokyo, Japan) at 20 × magnification. Lipid presence was quantified as the percentage of the area occupied by red-labeled fat droplets.

### Electrophysiological procedure

Rats were anesthetized, and a superior cervical ganglion (SCG), including the sympathetic cervical trunk and the postganglionic nerves, was dissected and transferred to a recording chamber containing Ringer-Krebs solution (composition in mM: NaCl, 136; KCl, 4; KH₂PO₄, 1; CaCl₂, 2; MgCl₂, 1; NaHCO₃, 12; glucose, 11), continuously gassed with 95% O₂ and 5% CO₂ (pH 7.4). The cervical sympathetic trunk was stimulated using glass suction electrodes with supramaximal pulses (10–20 V, 0.2 Hz, 0.1 ms) delivered by a Grass S88 stimulator (Grass Instrument, Quincy, MA, USA). Compound action potentials (CAPs) from the postganglionic internal carotid nerve were recorded using glass suction electrodes with an extracellular differential amplifier (DP-301, Warner Instruments, Hamden, CT, USA). The bandpass-filtered signals were digitized with a DAQ system and analyzed using a custom-made LabView-based program (National Instruments, Austin, TX, USA). To avoid saturation of the signal by the recruitment of all the postganglionic fibers during gLTP, nicotinic transmission was partially blocked (60–70%) by adding 100 μM hexamethonium to the bath [21]. LTP was produced by a train of supramaximal pulses of 0.1 ms duration, applied at 40 Hz for 3 s. CAP data were expressed as the ratio ΔR/R₀. The function f(t) = a e ⁻ ᵗ^/τ^¹ + c e ⁻ ᵗ^/τ^² was fitted to the data, and two parameters were used to compare gLTP: (1) gLTP duration, defined as the time required for CAPs to decay to 20% above baseline (f(t) = 0.2), and (2) gLTP magnitude, measured as the area under the potentiated response curve from t = 0 to the gLTP duration time.

### Immunohistochemistry

After rat perfusion (see Metabolic parameters section), the remaining SCG was dissected, unsheathed, post-fixed with paraformaldehyde, and cryoprotected in a 30% sucrose solution. The SCG was then frozen at −20°C and sectioned into 12 µm-thick longitudinal slices using a cryostat (Leica CM1520). Tissue sections were mounted on gelatin-coated Superfrost slides (Electron Microscopy Sciences). Ganglia were serially sectioned at 12 µm along the entire Z-axis, yielding approximately 40–45 sections per ganglion. A single section was randomly selected between sections 20th and 25th, corresponding to the central region, which we have previously characterized as exhibiting a consistent presence and distribution of VAChT- and GAD67-immunoreactive varicosities. Sections were washed in 0.1 M phosphate-buffered saline (PBS) for 10 minutes, then permeabilized and blocked for three hours at room temperature using PBS containing 0.3% Triton X-100 (PBS-Tx) and 10% normal donkey serum. Next, sections were incubated for 16 hours at room temperature in a humid chamber with primary antibodies diluted in a blocking solution (5% normal donkey serum, 5% bovine serum albumin, 0.3% Triton X-100): goat polyclonal anti-vesicular acetylcholine transporter (VAChT; Immunostar, Hudson, WI, USA, Cat # 24286, 1:200 dilution) and mouse monoclonal anti-L-glutamic acid decarboxylase (GAD67, the enzyme responsible for GABA synthesis; Millipore-Sigma, Burlington, MA, USA, Cat # MAB5406, 1:200 dilution). Following primary antibodies incubation, sections were washed twice for 15 minutes each in PBS-Tx and then incubated sequentially for two hours with secondary antibodies: donkey anti-mouse IgG CY3 (Jackson ImmunoResearch Lab Inc, West Grove, PA, USA, Cat # 715-165-151, 1:500 dilution), followed by donkey anti-goat IgG Alexa Fluor 488 (Jackson ImmunoResearch Lab, Cat # 715-585-150, 1:200 dilution). After secondary antibody incubation, sections were washed twice for 15 minutes each in PBS-Tx and mounted using fluorescence mounting medium (Dako, Carpinteria, CA, USA). Finally, sections were examined using a Nikon Eclipse E600 epifluorescence microscope (Nikon Corporation), equipped with appropriate filters for CY3 and Alexa Fluor 488, to select sections for analysis.

### Image acquisition and analysis

Images of selected tissue sections were acquired using a Nikon A1R+ laser scanning confocal head coupled to an Eclipse Ti-E inverted microscope (Nikon Corporation). The system was equipped with a motorized stage (TI-S-E, Nikon) and controlled via NIS-Elements C v.5.00 software. Tissue sections were examined using a PlanApo lambda 20X objective (N.A. 0.75). Single-plane images were sequentially captured using standard galvanometric scanners, with excitation wavelengths of 488 and 561 nm modulated by an acousto-optic tunable filter (AOTF). The parameters of pinhole size 3.9 µ, gain FITC: 32 a.u, TRITC: 21 a.u, contrast, and brightness used to capture and analyze images were kept constant between ganglia from control and experimental groups.

To identify specific labels, we used the Metamorph image analysis system (v. 7.5.6; Universal Imaging Corporation, Molecular Devices, Downingtown, PA, USA). Puncta were considered specifically labeled if their optical density (OD) exceeded the background level (defined as OD > background mean + 2 SD). A mask (template) was then generated to highlight the detected labeled varicosities. The number of pixels corresponding to each marker was quantified within labeled varicosities across the entire ganglion section. The area occupied by VAChT or GAD67 was expressed as a percentage of the total section area.

Colocalization of VAChT and GAD67 was assessed by calculating the proportion of varicosities coexpressing both markers relative to the total number of GAD67-immunoreactive varicosities. The percentage of varicosities expressing only GAD67 represented the degree of segregation.

### Statistics

For statistical analysis, a total of approximately 480 images were analyzed, 240 from each control and MS condition. For each ganglion, values were obtained from the analysis of 40 images that covered the entire section area. Images were captured sequentially using the galvanometric scanner of the confocal microscope. Data are expressed as mean values ± SEM and were analyzed with an independent two-tail Student’s t test, as indicated in the figure legends. The α level was 0.05.

## Results

### Metabolic syndrome is induced by high-sucrose diet

**Fig 1:**
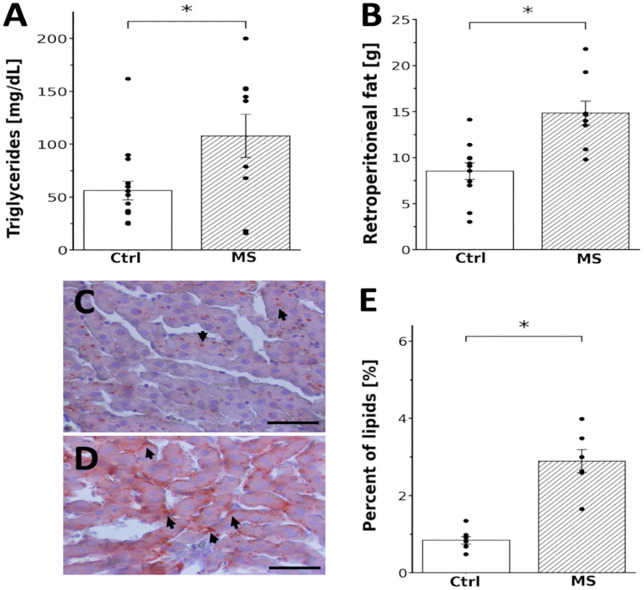
Serum triglycerides, retroperitoneal fat and hepatic lipids increased in HSD-fed rats (30% sucrose administered for 8 weeks). Graphs showing averages, SEM and individual values of serum triglycerides (A) and retroperitoneal fat **(B)**. Sucrose intake significantly increased 1.9-fold triglycerides in serum (56 ± 9 in control to 108 ± 22 mg/dL in HSD-fed rats. *t = −2.54, *df* = −1.07, *P* = 0.02), and 1.7-fold the presence of retroperitoneal fat (8.5 ± 1.0 in control vs 14.8 ± 1.0 grams in HSD-fed rats. *t = −3.86, *df* = −1.79, *P* = 0.001). C, D: Representative Oil-red-O stained liver sections, from control and HSD-fed rats. Red labeled lipids droplets (arrows) were more abundant in HSD-fed rats. Scale bar = 50 μm. E: Bar graph showing the percentage of area occupied by lipids in both control and HSD-fed rats (0.8 ± 0.1% in control to 2.9 ± 0.3%. * *t* = −6.34, *df* = −3.53, *P* = 0.02, n = 7).

To evaluate the physiological and metabolic effects of an 8-week high-sucrose diet (HSD), we measured several biomarkers. Clearly, serum triglycerides and retroperitoneal fat mass significantly increased in HSD-fed rats compared to controls. Serum triglyceride levels rose from 56 ± 9 mg/dL in control to 108 ± 22 mg/dL in HSD-fed rats (*t* = −2.54, *df* = −1.07, *P* = 0.02). Retroperitoneal fat mass increased from 8.5 ± 1.0 g in control to 14.8 ± 1.0 g in HSD-fed rats (*t* = −3.86, *df* = −1.79, *P* = 0.001; Fig 1A, B).

Additionally, we assessed hepatic steatosis as an indicator of metabolic dysfunction. HSD-fed rats exhibited a significant increase in lipid accumulation within the liver compared to controls. The liver area occupied by lipids expanded from 0.8 ± 0.1% in control to 2.9 ± 0.3% in HSD-fed rats (*t* = −6.34, *df* = −3.53, *P* = 0.02; Fig 1C–E).

### Ganglia from HSD-fed rats failed to express gLTP

In the SCG of Wistar rats, CAPs recorded from the postganglionic nerve reflect synaptic transmission between preganglionic varicosities and the soma and dendrites of ganglionic neurons. The contribution of bypassing fibers is negligible, as CAPs can be blocked by 90–95% with the cholinergic antagonist hexamethonium.

**Fig 2:**
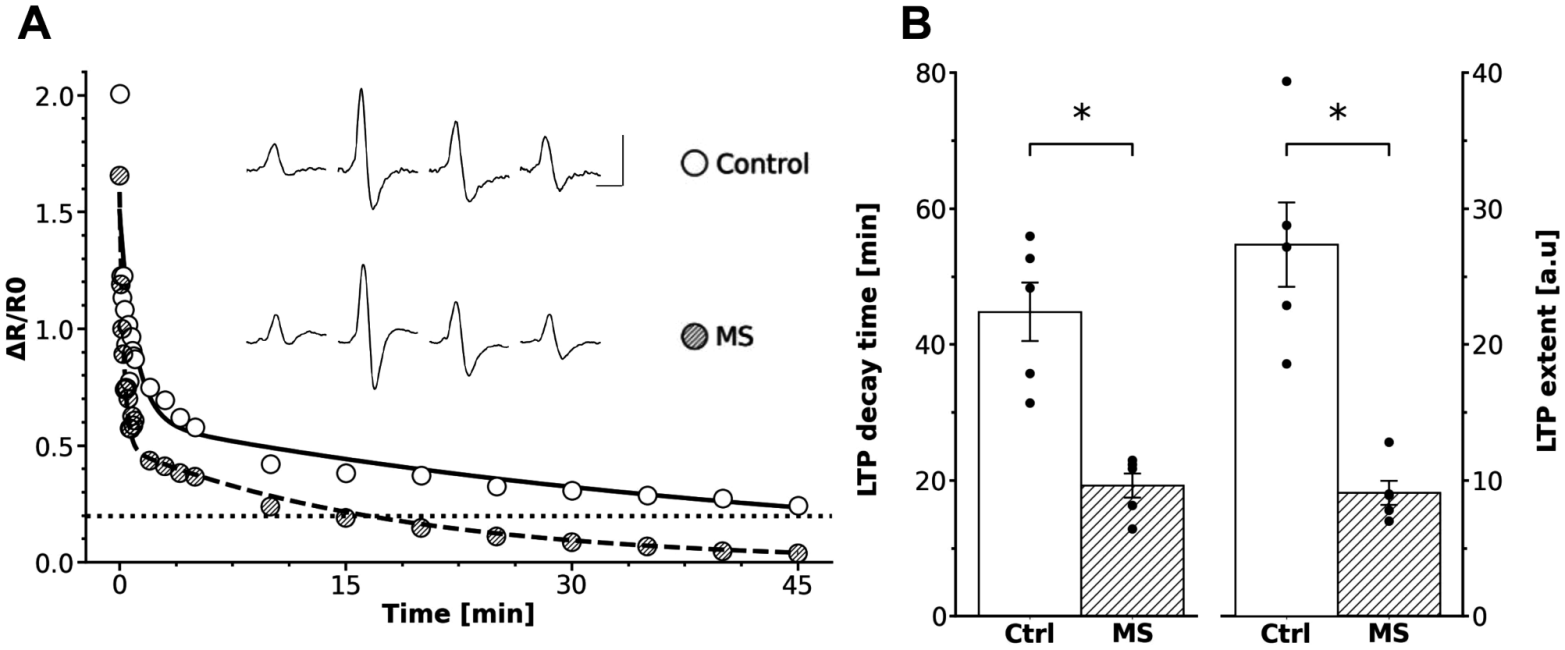
MS-associated dysautonomia impaired LTP expression in rat SCG. A: Time course of representative experiments showing synaptic potentiation of ganglionic transmission, expressed as ΔR/R0, recorded in control (○) and in HSD-fed rats (●). Dotted line indicates the LTP baseline value. Inset shows CAP records of control and HSD-fed rats, obtained before the train and at 5 sec, 5 min and 40 min post-train (scale bar represents 20 msec and 1 mV). B: Bar plots of analyzed LTP parameters (average ± SEM and individual values) showing that gLTP recorded in ganglia from HSD-fed rats was significantly smaller than in control rats. LTP decay time dropped from 45 ± 5 min in control to 19 ± 2 min in HSD-fed rats, whereas LTP extent fell from 27 ± 3 [a.u.] in control to 9 ± 1 [a.u.] in HSD-fed rats. Paired t-tests showed the differences between groups were significant (* *t* = 4.91, *df* = 3.11, *P* = 0.001 for decay time and *t* = 5.03, *df* = 3.18, *P* = 0.001 for extent; n = 5).

In the SCG of control rats, a high-frequency train of supramaximal pulses (40 Hz for 3 s) applied to the preganglionic nerve induced gLTP in the postganglionic nerve, characterized by a decay time of 45 ± 5 min and an extent of 27 ± 3 [a.u.]. In contrast, SCG from HSD-fed rats did not exhibit full gLTP. The same high-frequency stimulus train induced only transient post-train potentiation, which failed to reach the typical duration and magnitude of gLTP. In these rats, the decay time was significantly shorter (19 ± 2 min), and the extent was markedly reduced (9 ± 1 [a.u.]) compared to control SCG (*t* = 4.91, *df* = 3.11, *P* = 0.001 for decay time and *t* = 5.03, *df* = 3.18, *P* = 0.001 for extent; Fig 2).

### MS reduced expression of VAChT and segregation without affecting GABA occurrence

**Fig 3:**
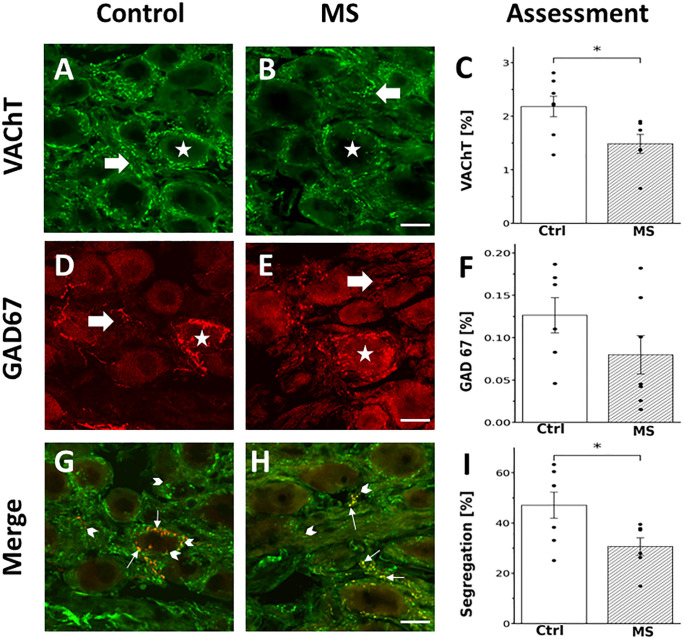
MS-associated dysautonomia reduced ACh expression and its segregation with GABA. Micrographs of SCG sections double immunolabeled for VAChT and GAD67 from control (A, D, G) and HSD-fed rats (B, E, **H)**, both VAChT and GAD67 positive varicosities were detected surrounding cell bodies (asterisk), more frequent in VAChT -IR or running between them (arrows). VAChT immunolabels were more abundant. G, H: Merged images of VAChT and GAD67 immunostained varicosities, GAD67 stain can be segregated (red dots, arrowheads) or colocalized with VAChT stain (yellow dots, small arrows). Bar graphs of mean, standard errors, and individual values depicting the percent of ganglionic area occupied by the varicosities positive to VAChT (C) and GAD67 (F) and the degrees of VAChT-GABA immunolabels segregation (I). MS-associated dysautonomia reduced VAChT immunolabel from 2.18 ± 0.19 of control ganglia to 1.48 ± 0.19% of total ganglionic area in HSD-fed rats (*t = 2.45, *df* = 1.35, *P* = 0.03). Conversely, GAD67 immunolabel was not altered, 0.12 ± 0.02% in control vs 0.07 ± 0.03 in HSD-fed rats (*t* = 1.37, *df* = 0.97, *P* = 0.2). Like VAChT presence, segregation of VAChT and GABA was decreased, 47.2 ± 5.6 in control rats to 30.7 ± 3.9 in HSD-fed rats, (* *t* = 2.43, *df* = 1.30, *P* = 0.04), n = 6. Calibration bar: 10 µm.

In both control and MS groups, we observed the presence and segregation of VAChT and GAD67, markers for ACh and GABA, respectively, in preganglionic varicosities within the SCG. Consistent with previous studies [5,22], GAD67-labeled varicosities were sparsely distributed throughout the ganglia, exhibiting two distinct patterns: (1) concentric varicose fibers surrounding some ganglionic principal neurons and (2) long interstitial fibers extending within the neuropil alongside neuronal cell bodies. In SCG, GAD67-positive varicosities occupied only 0.02–0.18% of the total ganglionic area (Fig 3D and 3E). In contrast, VAChT-immunoreactive (VAChT-IR) varicosities were widely distributed throughout the ganglion, covering 0.65–2.8% of ganglionic area, with most surrounding the soma of ganglionic neurons (Fig 3A and 3B). Double labeling revealed that some varicosities coexpressed VAChT and GAD67, while others expressed only one of the markers, confirming the coexistence and segregation of ACh and GABA. Segregation from VAChT was observed in 14% to 63% of all GAD67-positive varicosities (Fig 3G and 3H).

MS significantly altered VAChT expression and segregation, while GAD67 occurrence remained unaffected. Specifically, VAChT expression was significantly reduced from occupied area of 2.18 ± 0.19% in control to 1.48 ± 0.19% in MS rats (*t* = 2.45, *df* = 1.35, *P* = 0.03; Fig 3C). Similarly, segregation was significantly reduced, dropping from 47.2 ± 5.6% in control to 30.7 ± 3.9% in MS rats (*t* = 2.43, *df* = 1.30, *P* = 0.04; Fig. 3I). In contrast, GAD67 expression remained unchanged, with values of occupied area of 0.12 ± 0.02% in control versus 0.07 ± 0.03% in MS rats (*t* = 1.37, *df* = 0.97, *P* = 0.2; Fig 3F).

## Discussion

Our data confirm that rats consuming water with 30% sucrose for eight weeks developed a pathological condition resembling MS, since they show metabolic alterations including signs of hepatic steatosis. In addition to metabolic disturbances, HSD-fed animals displayed autonomic dysfunction characterized by a reduced synaptic plasticity, as they failed to express full gLTP, furthermore, they exhibited changes in the area occupied by ACh and its segregation within the preganglionic varicosities of the SCG, with both ACh presence and ACh-GABA segregation being diminished.

The failure of gLTP expression in HSD-fed rats aligns with previous findings showing reduced gLTP expression in models of autonomic dysfunction. Similar impairments have been reported in SHR [4,23], diabetic and hypertensive obese Zucker rats (OZR) [24], and in streptozotocin (STZ)-induced diabetes [25]. Several mechanisms have been proposed to explain this phenomenon. Alkadhi and colleagues suggested that in hypertensive and OZR rats, gLTP is absent due to an occlusion effect, where ganglionic transmission is already potentiated by sympathetic overactivity. In contrast, we proposed that the failure of gLTP in SHR results from increased GABAergic inhibition, as blocking GABA-A receptors restores gLTP expression [4]. Additionally, other studies suggest that increased reactive oxygen species (ROS) impair both basal synaptic transmission and gLTP expression in diabetic models [8,25].

In the STZ-induced diabetes model, impaired basal synaptic transmission and failure of gLTP were linked to reduced levels of brain-derived neurotrophic factor (BDNF) [25]. Consistent with these findings, it has been reported that BDNF is involved in the expression of gLTP in sympathetic ganglia [26] and also in hippocampal LTP [27,28]. BDNF is also known to play a role in the regulation of food intake [29], thus, low BDNF levels have been associated with obesity, insulin resistance, and other components of MS [30]. In an HSD-fed rat model of MS, decreased RNA and protein levels of BDNF have been reported [31]. Based on this evidence, we hypothesize that the impairment of gLTP expression in HSD-fed rats may be due to reduced ganglionic BDNF levels or dysfunction in related signaling pathways. Measuring ganglionic BDNF levels would help to test this hypothesis.

Concerning alterations in neurotransmitter presence and distribution, we found that MS-associated dysautonomia led to a reduction in the ganglionic area occupied by ACh and a decrease in ACh-GABA segregation, while GABA presence remained unchanged. A decrease in ACh expression suggests a reduction in ganglionic synaptic transmission. Similarly, we have previously shown that decreased ACh-GABA segregation enhances GABAergic inhibition [6,22]. Both findings indicate a reduction in sympathetic function at the peripheral level.

There are conflicting results regarding the type of autonomic dysfunction associated with MS. Some studies have reported signs of sympathetic overactivity, including increased heart rate, elevated NE levels in plasma and urine, and heightened efferent muscle sympathetic nerve activity [14,16,17,32,33]. In contrast, other studies conducted at the same peripheral level as ours have shown synaptic transmission depression in the SCG and celiac ganglion of STZ-treated mice [8,18]. This synaptic depression correlates with reduced sympathetic nerve activity, as evidenced by decreased heart rate and impaired thermoregulation [8]. These effects have been linked to nicotinic ACh receptor inactivation [8]. It is likely that, in our experimental model of MS, rats also experience cholinergic receptor inactivation in the SCG, potentially contributing to the reduction in gLTP expression. Beyond this postsynaptic effect, reduced ACh presence and impaired ACh-GABA segregation may also represent presynaptic factors contributing to diminished synaptic transmission.

Finally, these considerations are in direct relation with the known SNS ability to act in a regionally differentiated manner [34,35]. For instance, the chemoreceptor reflex induces a pressor response in the forearm vasculature accompanied by bradycardia [34]. Similarly, an increase in central sympathetic neural outflow elevates heart rate and blood pressure while reducing sympathetic vascular tone in adult males [36]. Accordingly, in our MS model, sympathetic activity may increase in some regions while decrease in others, such as in the sympathetic ganglion.

## Conclusions

MS-associated dysautonomia affects synaptic plasticity, leading to impaired LTP expression and altered neurotransmitter dynamics, including reduced ACh presence and a reduction in the degree of ACh-GABA segregation. Lower ACh availability and decreased ACh-GABA segregation may reduce ganglionic cholinergic transmission and contribute to gLTP impairment. These changes indicate autonomic dysfunction at peripheral level.
