# Correction: Long-term potentiation and neurotransmitters expression and segregation are altered in the Metabolic Syndrome-associated dysautonomia

**DOI:** 10.1371/journal.pone.0353741

**Published:** 2026-07-13

**Authors:** Diana Elinos, Fernanda Veladiz-Gracia, Constanza González-Sierra, Angel Rubio-Galicia, Fredy Cifuentes, Miguel A. Morales

There is an error in the references 13 and 24. The correct references are:

13. Schaffer JD, Sun J, Alkayed NJ, Traystman RJ, Hurn PD. Sex differences in sympathetic neural control in rats after stroke. Front Physiol. 2014;5:284. https://doi.org/10.3389/fphys.2014.00284

24. Alzoubi KH, Aleisa AM, Alkadhi KA. Expression of gLTP in sympathetic ganglia of obese Zucker rats in vivo: Molecular evidence. J Mol Neurosci. 2008;35(3):297–306. https://doi.org/10.1007/s12031-008-9050-1
